# In vitro bioproduction and enhancement of moscatilin from a threatened tropical epiphytic orchid, *Dendrobium ovatum* (Willd.) Kraenzl

**DOI:** 10.1007/s13205-021-03059-1

**Published:** 2021-11-22

**Authors:** Ipsita Pujari, Abitha Thomas, Padmalatha S. Rai, Kapaettu Satyamoorthy, Vidhu Sankar Babu

**Affiliations:** 1 Department of Plant Sciences, Manipal School of Life Sciences, Manipal Academy of Higher Education, Manipal, Udupi, Karnataka 576104 India; 2 Department of Biotechnology, Manipal School of Life Sciences, Manipal Academy of Higher Education, Manipal, Karnataka India; 3 Department of Cell and Molecular Biology, Manipal School of Life Sciences, Manipal Academy of Higher Education, Manipal, Karnataka India

**Keywords:** *Dendrobium ovatum*, Elicitor, In vitro cultures, Moscatilin, Precursor

## Abstract

Moscatilin, a bibenzyl derivative (stilbenoid), mostly found in one of the largest genera of Orchidaceae; *Dendrobium* has many therapeutic benefits. Its function as an anticancer agent has been widely demonstrated through many research investigations. However, the compound has not been produced in vitro to date. The present study highlights the development of cultures viz., seedling generation, callus induction and callus regeneration (transformation of callus into plantlets). These cultures were devised to conserve the threatened tropical epiphytic orchid species, *Dendrobium ovatum* and identify their potential towards moscatilin bioproduction in vitro. Among the three culture platforms, callus-derived plantlets could yield high moscatilin when treated with l-Phenylalanine as a precursor. Tissue differentiation was found to be indispensable for the high production of this polyphenol. These cultures also offer potential commercial benefits as they can serve as appropriate platforms to decode moscatilin biosynthesis and other significant bibenzyl derivatives. Elicitors, such as chitosan, salicylic acid, and methyl jasmonate, were found, causing an enhancement in moscatilin content in the cultures. The seedlings obtained can serve towards ecorestoration and preservation of the studied species. Callogenesis was useful in plantlet regeneration, as callus-derived plantlets could be utilized for the enrichment and commercial scale-up of moscatilin-like chemicals.

## Introduction

A plant in vitro system serves as a viable technique for the production of natural products. The tissue culture procedures are excellent biotechnological approaches for the biosynthesis of high-value phytochemicals, as the natural habitats are continually perishing due to climate change and rapid industrialization (Marchev et al. 2020). In vitro plant cultures have proven continuously their efficiency towards manufacturing uncommon and improved-quality bioactive secondary metabolites as they equip advanced and regulated development that is free from environmental interruption and is also season-independent. These approaches generate distinct tissue types under various differentiation modes, activating the biosynthetic routes producing targeted plant chemicals. The in vitro culture platforms are meant to conserve species (especially if threatened) and restore them to their congenial/natural habitat. They provide a stage to screen the metabolites, study their molecular precursors and molecular targets using suitable analytical techniques/bioassays (Ochoa-Villarreal et al. 2016). In this context, the role of elicitors and precursors is essential, as they cause more metabolite accumulation through activation of defence responses against stress or the inclusion of a source/intermediate organic compound of the relevant pathway to obtain the final product, respectively (Espinosa-Leal et al. 2018). Plant-derived metabolites are biologically potent with an extensive spectrum of targets as well as actions, and they are found to be mostly safe. Phytodrugs exhibit ‘metabolite likeness,’ which indicates that they are target-specific, and they transport molecules quite efficiently into their intracellular activity sites (Marchev et al. 2020). A majority of the drugs presently approved by the Food and Drug Administration (FDA) and the European Medicines Agency (EMA) are plant-derived small molecules, the most successful ones being anticancer agents (Thomford et al. 2018). Plant natural products are typically present in minimal quantities, and the requirement of plentiful harvesting materials to fulfil their current commercial demand is environmentally reproachful and unfeasible (Marchev et al. 2020). The trend in research on plant-based drugs has always focused on identifying active principles and their therapeutic effectiveness rather than the cultivation of plant species that accommodate them (Salmerón-Manzano et al. 2020). A large group of significant plant natural products with distinctive chemical features in chiral centres, complex aromatic rings, number and ratios of heteroatoms are still waiting to be discovered and produced (Marchev et al. 2020).

One such compound which has not been produced in vitro so far is moscatilin, also known as Dendrophenol or, 4,4ʹ-dihydroxy-3,3ʹ,5-trimethoxybibenzyl (molecular formula: C_17_H_20_0_5_, molecular weight: 304.34 g/mol). The compound was first isolated from the wild endangered orchid, *Dendrobium moscatum* (Majumder and Sen 1987). Orchidaceae is the most evolved and diverse family of angiosperms comprising over 27,000 species, categorized under ~ 1000 genera and distributed over all the continents except Antarctica (Wraith et al. 2020). Orchids contribute substantially to the global cut flower trade because of their ornamental values, and their medicinal uses are also known in Chinese, South Asian Ayurvedic and African traditional medicine along with North American folk medicine (Hinsley et al. 2018). Orchidaceae is one of the world’s most threatened plant families, with greater than 600 species indexed as threatened under the IUCN Red List, and the reasons behind this are their innate rarity, definite conservation method and anthropogenic threats (Wraith et al. 2020). Orchids, just like other plants, stock a large group of phytoconstituents, such as alkaloids, bibenzyls, stilbenes, fluorenones, phenanthrenes, coumarins, that hold their curative properties (Sut et al. 2017). *Dendrobium* is one of the largest genera of Orchidaceae with ~ 1400 species, and its medicinal value, such as antibacterial, neuroprotective, antidiabetic, immunomodulation, antiangiogenic properties, have been widely reported, including their antipyretic, analgesic and tonic activities in Traditional Chinese Medicine (TCM) (Teixeira da Silva and Ng 2017). The species under this genus have high economic worth in the horticulture and floriculture industry as hybrids, potted orchids and cut flowers. This *Dendrobium* genus of orchid is massively traded, and it occupies Annex II of the Convention on International Trade in Endangered Species of Wild Fauna and Flora (CITES). Though all the three classes of secondary metabolites (alkaloids, phenolics and terpenoids) exist in them, phenolic compounds serve immense medicinal benefits, and in recent years, they are much studied. Bibenzyl derivatives are one of these essential phenolics, and these were found to exhibit multiple therapeutic actions such as antitumor, anti-inflammatory and antioxidant properties (He et al. 2020). Approximately 100 *Dendrobium* species are reported from India, and out of them, *Dendrobium nodosum*, *Dendrobium plicatile* and *Dendrobium macraei Lindl.* have been extensively used in the Ayurvedic system of medicine as ‘Jivanti’, specified in Charaka Samhita (Pal et al. 2020). A less-studied species, namely *Dendrobium ovatum* (Willd.) Kraenzl is an epiphytic threatened herbaceous tropical orchid, that is found to be endemic to the Western Ghats and their spillover regions in India. Commonly it is referred to as ‘green-lipped *Dendrobium*’ because of the prominent green centre in off-white/cream-colored flowers. Juice of this fresh plant has medicinal uses such as laxative, stomachic, liver tonic, antispasmodic and carminative. Also, this species is used as a substitute for Jivanti in Ayurveda (Khare 2007). Like any other orchids in the tropics, the seed propagation of *D. ovatum* is also at the mercy of nature. These seeds are mostly non-endospermous or, with very little endosperm and they lack nutritional reserves. Even though their capsules produce hundreds of minute seeds, most of them fail to germinate in the wild, as the germination of filamentous seeds needs the support of specialized ectomycorrhizal ascomycetes or basidiomycete partners in the form of symbiosis for organic matter supplementation (Cardoso et al. 2020). Absence of fungal associates, deficient moisture content, advancing deforestation and urbanization, all these contribute to protracted seed germination diminishing the expansion of their population in the wild. This is the reason in vitro propagation of *Dendrobium*-like-orchids through an asymbiotic approach stands essential, that can produce clonal plantlets all year round accomplishing the market needs. It has also been observed that the in vitro orchid seed germination rates are more than 70%, which seldom exceed 5% in ex vitro natural habitat conditions (Cardoso et al. 2020). The seeds are well adapted to be quiescent during summer and their intrinsic dormancy breaking ability is not well studied. Very little has been explored about the medicinal properties of *D. ovatum* as well. Studies on *Dendrobium* orchids have indicated that the most crucial bibenzyl active principle isolated from them is, ‘Moscatilin’. This compound exhibits anti-inflammatory, antioxidant, antiplatelet aggregation and antimutagenic activities (Miyazawa et al. 1999; Chen et al. 2019). Largely, moscatilin has been identified as an anticancer therapeutic agent resulting in apparent cytotoxicity at non-toxic concentrations against multiple cancers, such as esophageal (cell line: CE81T/VGH), head and neck (cell line: FaDu), pancreatic (cell line: PANC-1), lung (cell line: NCI-H23), breast (cell line: MDA-MB-231), and colorectal (cell line: HCT-116) through apoptosis and anti-metastatic action (Kowitdamrong et al. 2013; Chen et al. 2019; Lee et al. 2020). The first research report demonstrating moscatilin as a potential anticancer agent came in 2003, where its cytotoxic effect was recognized against the cancer cell lines derived from diverse tissue origins viz., lung, stomach and placenta (Ho and Chen 2003). Lately, the significance of moscatilin against melanoma (cell line: A375), through induction of apoptosis and necrosis has also been described (Cardile et al. 2020). Many research studies have reported moscatilin in several wild species of *Dendrobium*, such as *Dendrobium loddigesii*, *Dendrobium nobile*, *Dendrobium pulchellum* (Miyazawa et al. 1999; Kowitdamrong et al. 2013; Chen et al. 2019) extensively. But as this genus is largely threatened, to conserve the species, notably *D. ovatum,* it is essential to raise tissue culture platforms. As the in vitro plant propagation offers significant advantages, such as yield stability, shorter production cycles (in comparison to the wild plants) and enhanced biosafety (due to absence of genetic or environmental contaminations) through current good manufacturing practices (cGMP), it serves essential for this study (Marchev et al. 2020). These tissue cultures can be used efficiently to study the presence of moscatilin, strategise methods to enhance the compound in the species and ecorestore them in natural habitats. Many approaches, such as optimization of culture medium, elicitation and precursor techniques, plant cell immobilization, permeabilization, selection of elite cell lines, have been used towards the enhancement of plant natural products (Ochoa-Villarreal et al. 2016; Espinosa-Leal et al. 2018). The present study is based on establishing three tissue culture protocols that include (1) seed germination (2) callogenesis and (3) plantlet regeneration from calluses, evaluating their abilities to yield Moscatilin in vitro. Out of these three, the high moscatilin yielding culture system was chosen for both precursor and elicitor treatments and the yield was compared with the whole *D. ovatum* plants collected from the wild habitat. This research is the first to establish three different tissue culture protocols with distinct differentiation patterns towards the production of moscatilin in vitro in economically and medicinally potent threatened orchid species, *D. ovatum*.

## Materials and methods

### Chemicals and materials

Capsules of *Dendrobium ovatum* were collected from the Hebri region (a spillover zone of Western Ghats, Karnataka), India. Authentication of the specimen was done by a distinguished taxonomist Dr K. Gopalakrishna Bhat, and the voucher specimens were kept at the herbarium of Manipal School of Life Sciences (MSLS), Manipal, Karnataka, India. Tween-20 and Mercuric chloride were purchased from HiMedia Laboratories Pvt. Limited, Mumbai, India, and Qualigens. All the nutrient medium ingredients (MS Macroelements, Zeatin, Sucrose and Plain Nutrient Agar, 6-BAP, 2,4-D) for the tissue cultures were purchased from HiMedia Laboratories Pvt. Limited, Mumbai, India. Micro salts and vitamins were manually prepared as stock solutions and were added to the medium. Moscatilin (HPLC > 98% purity) in the form of off-white crystal was purchased from Chengdu Biopurify Phytochemicals, Ltd., Sichuan, China. Solvent methanol (99.8%) that was used for the extraction and Reversed-Phase High-Performance Liquid Chromatography (RP-HPLC) was of HPLC and UV Spectroscopy grade and was purchased from Sisco Research Laboratories (SRL), Pvt. Ltd., Mumbai, India. The precursor compounds used for the study were l-Phenylalanine and l-Tyrosine (extra pure CHR, 99%, SRL, Pvt. Ltd., Mumbai, India). The elicitors used for the study were Chitosan (extra pure, SRL), Methyl jasmonate (Sigma-Aldrich, Merck, USA), Salicylic acid (99% pure, SRL) and Yeast extract (for bacteriology, SRL). RP-HPLC analysis was performed using the Agilent ZORBAX Eclipse Plus C18 column (250 mm × 4.6 mm, 5 µm pore size). The solvents used during RP-HPLC viz., Acetonitrile (ACN) was 99.9%, HPLC and UV Spectroscopy grade from SRL and Trifluoroacetic acid (TFA) was LC–MS grade, from Merck.

### Plant material and growth conditions in vitro

The whole wild plant of *D. ovatum* was collected during the Summer and Monsoon seasons in 2017, 2018 and 2019. For in vitro propagation, *D. ovatum* capsules were taken, and they were first subjected to surface sterilization with 10% of Tween-20 detergent solution for 10 min. This was followed by washing under running tap water for 30–40 min. After that, capsules were washed through autoclaved distilled water twice. Then they were surface-sterilized using autoclaved double distilled water two times inside the horizontal laminar airflow (LAF) cabinet. The fruits were then treated with 0.1% disinfectant Mercuric chloride solution for 10 min. This was followed by five series of rinsing in autoclaved double distilled water to remove traces of disinfectant. Post-wash capsules were blotted dry on sterilized filter papers for the removal of water traces. Then they were split longitudinally using a sterile surgical blade, and the filamentous seeds of *D. ovatum* were spread onto half-strength Murashige and Skoog basal culture medium (Murashige and Skoog 1962) containing Macroelements (4.23 g [gm] of dehydrated macroelements per litre) and supplemented with 1 mg/l Zeatin, 2% (*w/v*) Sucrose, and the pH was adjusted to 5.8. For the solidification of the medium, 0.8% (*w/v*) Agar was used, and the culture medium was autoclaved at 121 °C, 15 psi for 15–20 min. Culture incubation conditions were set at a temperature of 25 ± 2 °C with a 12-h photoperiod maintained by fluorescent lights (Philips India Ltd., Mumbai, India) with photosynthetic photon flux density (PPFD) 50 μmol m^−2^ s^−1^ in a growth room.

### Seed culture

For seed culture, half-strength Murashige and Skoog (with macroelements, microelements, vitamins) agar-based solid medium was used, that was supplemented with 1 mg/l Zeatin and 2% Sucrose. This medium was found useful for the generation of Protocorms and Protocorm-like bodies (PLBs), the developmental stages that usually get formed during in vitro culture of any orchid. Therefore this medium was denoted as ‘protocorm/PLB initiating medium (PPIM)’. These protocorms and PLBs were subcultured in fresh half-strength MS media supplemented with 2 mg/l of 6-BAP along with 1 mg/l Zeatin, 5% Sucrose with pH 5.8, towards the development of young plantlets. Later these young plantlets were shifted to PPIM again to develop plantlets with roots and shoots during subsequent subcultures.

Every subculture was carried out within every 20 days interval. And during each subculture, the respective plant growth stages were transferred to different culture bottles with freshly prepared MS nutrient media with required additives for further multiplication. All the developmental stages of *D. ovatum *in vitro were observed and identified under a Stereo Zoom Microscope (‘Motic’), equipped with the software ‘Motic images plus 2.0’. These microscopic images were captured in the form of stereomicrographs. Biomass estimations (fresh and dry weights) of seed-derived young plantlets/seedlings were carried out and compared within a single subculture duration (20 days). For the measurement of dry weight, young plantlets were freeze-dried at a temperature of − 50 °C, for approximately 48 h. Representation of fresh and dry weights of young plantlets was in the form of gram (gm). Five different tissue culture bottles containing the proliferated young plantlets were considered for both fresh and dry weight estimations and comparisons. Along with the biomass estimations, bioproduction of Moscatilin was estimated in well-proliferated young plantlets. Two different set-ups in the form of non-subcultured and subcultured young plantlets were also built for a 100-day study to observe the pattern of Moscatilin bioproduction. For this investigation, the young plantlets with maximum proliferation were considered and then they were studied for another 100 days towards Moscatilin bioproduction. During this study, the non-subcultured plantlets were not subjected to subculturing for 100 days and then subcultured plantlets were subcultured within every 20 days interval. These two groups of cultures were maintained with five tissue culture bottles with the young plantlets in each case. In two different trials, inter-day (on 0th, 20th, 40th, 60th, 80th and 100th day) and intra-day (in 0, 3, 6, 9, 12 and 15 h), variations were estimated and compared. The main objective behind this was to check the effect of steady subculturing towards the accumulation of moscatilin in young plantlets.

### Callogenesis

The Protocorms and PLBs served as explants for the callus formation in *D. ovatum*. The explants were shifted from PPIM to half-strength MS basal medium augmented with the combination of an auxin, i.e., 1 mg/l 2,4-D and a cytokinin i.e., 0.5 mg/l 6-BAP along with 0.5 mg/l Zeatin hormone, 3% (*w/v*) of Sucrose. pH of the medium was fixed to 5.8 with 1 M NaOH or 1 N HCl, solidified with 0.8% (*w/v*) Agar and autoclaved at 121 °C, 15 psi for 15–20 min. Culture incubation conditions required for the callus formation were the same as mentioned before. Subculturing was repeated continuously at every 20 days interval to visualize the efficacy of hormones on the texture and growth of calluses. All callus types formed were identified and captured in the form of stereomicrographs. Callus growth was observed for several weeks/months to observe the different types of calluses with ample biomass. After a great proliferation was achieved, biomass measurements (fresh and dry weights) of all the callus types within a single subculture duration was also determined and correlated. During these estimations, five different tissue culture bottles corresponding to each callus type were considered. Callus biomasses in the form of fresh and dry weights were expressed in gm. Bioproduction of moscatilin was checked in every well-proliferated callus type. Five tissue culture bottles with the growing calluses were chosen for this estimation.

### Plantlet regeneration from calluses

For the formation of callus-derived plantlets, embryogenic, non-embryogenic and organogenic callus types were transferred to half-strength MS medium supplemented with 2 mg/l 6-BAP along with 1–2 mg/l Zeatin and 5% Sucrose (termed as ‘regeneration medium’). The pH was fixed to 5.8 with 1 M NaOH or 1 N HCl, solidified with 0.8% (*w/v*) Agar and autoclaved at 121 °C, 15 psi for 15–20 min. Culture incubation conditions in the growth room were the same as before, and subculturing was done at every 20 days interval. Further growth in plantlet regeneration was observed and captured in the form of stereomicrographs. When they attained much proliferation, biomass measurements (fresh and dry weights) of both types of callus-derived plantlets were also estimated and compared within a single subculture duration. During these estimations, five different tissue culture bottles with the grown regenerative plantlets were considered. The biomasses of these callus-derived plantlets in the form of fresh and dry weights were expressed in the form of gm. In the well-grown regenerative plantlets, Moscatilin bioproduction was estimated for which five tissue culture bottles with the growing plantlets were chosen.

### Elicitor treatment

For elicitor treatment, the stage that was yielding high Moscatilin content was chosen. Four elicitors viz., (biotic elicitors—Methyl jasmonate, Yeast extract and Chitosan) and (abiotic elicitor—Salicylic acid) were used for the test cultures. The elicitor study involved MS basal medium in the liquid (without Agar) form, and it was autoclaved at 121 °C, 15 psi for 15–20 min. A uniform concentration of each elicitor (50 mg/l) was added to the half-strength MS media, where the tissues were inoculated, and culture incubation conditions were the same as prior. After 12 h of treatment, the extracts were prepared, concentrated and stored at − 80 °C till they were used for RP-HPLC analysis to inspect the effect of each elicitor towards in vitro bioproduction of moscatilin*,* identifying the best elicitor. Controls (without any elicitor) and tests (with elicitor) were maintained with five tissue culture bottles under each, containing 1 gm of plant tissue. This was done to observe if any enhancement in the quantity of moscatilin after the addition of elicitors.

### Precursor feeding

For this analysis, the stage that was yielding high Moscatilin content was chosen. The precursor feeding study involved MS basal medium in the liquid (without Agar) form, and it was autoclaved under the same conditions as before. l-Phenylalanine and l-Tyrosine were chosen as precursors with concentrations 1, 10 and 100 µM. Controls (without any precursor) and tests (with precursor) were maintained with five tissue culture bottles under each, with 1 gm of plant tissue. Culture incubation conditions in the growth room were set at the same conditions as prior. The treatment was for 12 h, and after 12 h, the extracts were prepared, concentrated and stored at − 80℃ till they were used for RP-HPLC analysis towards the identification and quantification of Moscatilin in them. The compound yield was compared between the control and test systems to check the precursors' role and identify the efficient precursor out of l-Phenylalanine and l-Tyrosine.

### Low-temperature based extraction method

1 gm of fresh *D. ovatum* whole plant collected from the wild and 1 gm of tissue from various in vitro cultures [i.e., seed-derived young plantlet (non-subcultured and subcultured), callus, regenerative plantlet along with the control and test samples involved in “Precursor feeding” and ‘elicitation’] were processed separately. First, all the tissues were frozen in liquid nitrogen and ground to a fine powder using a mortar and pestle. Then the plant powder was dissolved in a minimum volume (~ 1 ml) of the solvent Methanol for extraction, and the grinding continued till obtaining the powder in its finest form. This was followed by the addition of ~ 5 ml of Methanol to the powdered extract. Then the extract was subjected to pulse-sonication (Sonics Vibra-cell, USA) at 40 W for around 45 min with the intermittent pulse of 30 s (on/off cycle). All through, the extract samples were kept on ice. Sonication was followed by centrifugation at 4500 RCF for 30 min at 4 °C. The clear supernatant was concentrated using the lyophilization method (Thermo Fisher Scientific Heto PowerDry LL1500 Freeze Dryer). These concentrated extracts were used for RP-HPLC and Mass Spectrometry (MS) analyses to identify and quantify Moscatilin in all the studied systems.

### Quantification of Moscatilin from wild and in vitro samples of *D. ovatum* through RP-HPLC

Both wild whole plant of *D. ovatum* [(total 10 in number per each year): 5 (Summer) + 5 (Monsoon)] and in vitro samples of the study were processed through RP-HPLC (Waters® Alliance e2695 Separations Module), furnished with an autosampler, degasser, ultraviolet detector (Waters® 2487 Dual Wavelength Absorbance Detector) set at 281 nm wavelength and a dual pump. Stock solutions of Moscatilin standard were prepared in 99.8% pure HPLC grade methanol at a concentration of 5 mg/ml. A set of working standard solutions (0.5, 1.0, 1.5 and 2.0 mg/ml) was also freshly prepared from the stock Moscatilin solution using the solvent Methanol. The standard solutions and the study samples were managed with care to avoid degradation through light and air exposures. Here, the optimal separation was achieved by isocratic elution with a mobile phase consisting of the solvents, ACN—eluent ‘A’ and 0.001% TFA in milli-Q water—eluent ‘B’ in the ratio of 40:60 (*v/v*). The solvents were degassed by sonication for 15 min at room temperature before use. Chromatographic separation was achieved on a C18 column at 30 °C. The pressure was maintained at around 1200–1300 psi, and the flow rate was 1 ml/minute. The injection volume (for injection into the HPLC instrument for quantification) of 10 μl was used for the Moscatilin standard, control and the test samples. Run time was fixed to 30 min. While run, retention time (RT) and peak area for Moscatilin standard and blank were noted. Identification of Moscatilin was analysed in all the test samples based upon its retention time by juxtaposing the elution time of the compound in test and the same in the authentic standard. Quantification of Moscatilin was carried out using the respective peak areas obtained in comparison with the peak area of Moscatilin standard. Acquisition and data analysis of RP-HPLC were done using Waters® Empower™ 1 Software. All quantitative estimations were performed in replicates (*n* = 5), and the yields of Moscatilin were represented as µg/g equivalents of the dry weight of *D. ovatum* plant tissue extract. Overall, through RP-HPLC, the influence of Summer and Monsoon seasons (during the collection years 2017, 2018 and 2019) on the production of Moscatilin, which was extracted from the whole wild plants of *D. ovatum* was studied. This yield from the wild systems was compared with the same from all the in vitro systems of the study.

### Targeted metabolite profiling of Moscatilin through mass spectrometry

Accurate identification of Moscatilin was performed through Electrospray Ionization Quadrupole-Time-of-Flight Mass Spectrometry (ESI-QTOF-MS) in *D. ovatum* young seedlings, calluses and callus-derived plantlets that were generated in vitro. The analysis was done using Agilent 6520 Accurate Mass Q-TOF LC/MS. Positive ion mode was used along with the following conditions; capillary voltage 19 V, spray voltage 5.0 kV, capillary temperature 300 °C, sheath gas flow rate at 40 (arbitrary units), the auxiliary gas flow rate at 20 (arbitrary units) and tube lens offset 40 V. The mobile phase used here was the same as the ones used for RP-HPLC. The full-scan EI mass spectra were acquired in the *m/z* range of 50–400. Before processing the lyophilized tissue extracts through a mass spectrometer, they were dissolved in a minimum volume (~ 10 µl) of the solvent methanol, and the clear supernatant from the top was taken for the analysis. An injection volume of 10 μl was used for Moscatilin standard, blank and the test samples, that were injected into the carrier gas stream of electrospray source. Injections were performed in replicates for all the sample types. All the information collected through the mass spectrometer data system as mass spectra were retrieved. Identification of Moscatilin in the test seedling, callus and callus-derived plantlet samples was based upon the comparison of elution time and mass spectrum with that obtained from the authentic Moscatilin standard.

### Statistical analysis

The identification and quantitative analyses through RP-HPLC and MS were performed in replicates. All the data related to Moscatilin quantification were represented in the form of mean ± standard deviation (SD) of independent replications of randomized experiments. One-way ANOVA with multiple comparisons was performed to obtain the value of significance between the groups, where values with *P < 0.05, **P < 0.01, ***P < 0.001 and ****P < 0.0001 were considered statistically significant and ‘ns’ depicted non-significant at the 0.05 probability level. In “Precursor feeding” and elicitation studies, the relative increment in Moscatilin yields in test samples was compared to that of the corresponding controls. All the statistical analyses were carried out through GraphPad Prism 8.0.

## Results

### Hypergeneration of plantlets and biomass comparison

**Fig. 1:**
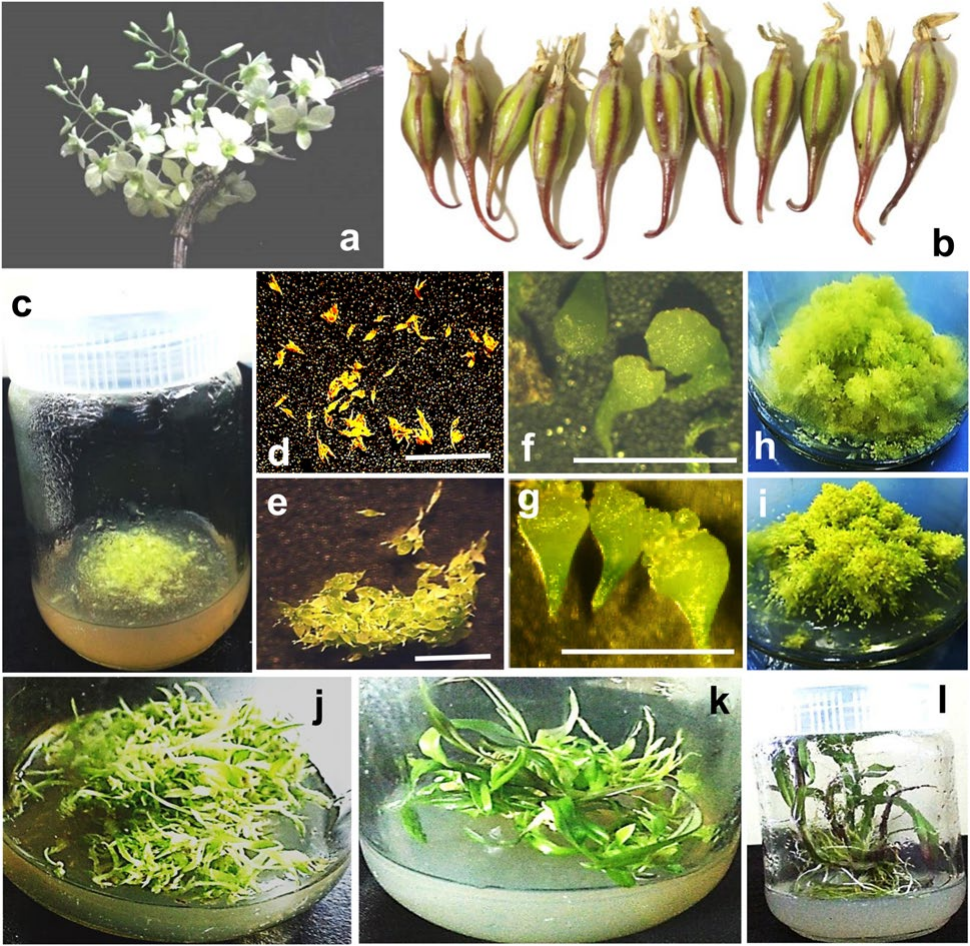
Habit of *Dendrobium ovatum* after attaining the reproductive phase (**a**). Capsules (fruits) of *Dendrobium ovatum* (**b**). Seeds from the capsules were inoculated onto PPIM, shown on the 0th day of the culture (**c**). Stereomicrographs of seeds, shown on the 0th and 5th day of the culture (**d**, **e**). Stereomicrographs of protocorms on the 20th day of seed culture (**f**), and protocorm-like bodies on the 40th day of seed culture (**g**). Hypergenerating protocorms (**h**), and protocorm-like bodies (**i**) on the 60th day of seed culture. Plantlets developed from the PLBs (**j**), and developed from the protocorms (**k**), shown on the 100th day of seed culture. Plantlets with well-developed shoots and aerial roots with velamen (**l**) on 160th day of seed culture, ready for acclimatization followed by ecorestoration in another 20 days (Scale bar = 1 cm)

**Fig. 2:**
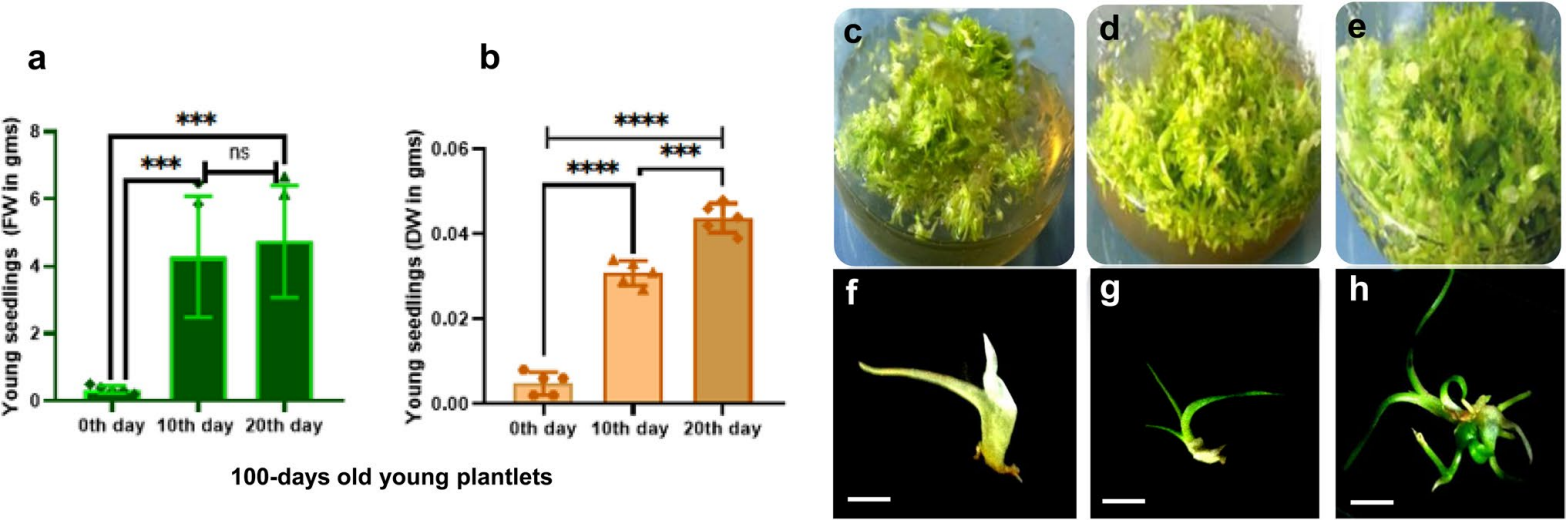
Graphical representations of both Fresh Weight (FW) and Dry Weight (DW) in well-proliferated 100 days old young plantlets, that were obtained through seed culture (**a**, **b**). Each group comprised five replicates. One-way ANOVA with multiple comparisons was performed to obtain the value of significance between the groups. Plantlet hypergeneration on the MS medium during a single subculture duration, shown in every 10 days interval; on 0th day (**c**), 10th day (**d**), and 20th day (**e**). Stereomicrograph of a seedling on the 0th day (**f**), 10th day (**g**), and 20th day (**h**) during a single subculture duration (Scale bar = 1 cm)

Through seed culture, the entire life-cycle of *D. ovatum *in vitro was staged in the half-strength Murashige and Skoog medium. For the seed culture, first, the capsules (Fig. 1b) were collected from *D. ovatum* plant (Fig. 1a). Then, the minute seeds from the capsules were dusted in a single tissue culture vessel containing PPIM (Fig. 1c) and the microscopic images of the seeds on the 0th day of seed culture were captured in the form of stereomicrographs (Fig. 1d). These seeds got swollen towards the 5th day (Fig. 1e) and developed into spherules in 10 days. These spherules gradually got converted into protocorms at the end of the 20th day (Fig. 1f). Every protocorm gave rise to protocorm-like bodies (PLBs) and multiplied rapidly by the 40th day of the culture (Fig. 1g). Formation of PLBs in the culture indicated ‘direct somatic embryogenesis’ in half-strength MS medium. Hypergenerating protocorms and PLBs were visible clearly towards the 60th day of seed culture (Fig. 1h, i). At this stage, the young seedlings were transferred to the half-strength MS media with both Zeatin and 6-BAP and high Sucrose (5%) compared to the low level of Sucrose (2%) PPIM. This combination resulted in the formation of an enormous number of young plantlets (hypergeneration) both from protocorms and PLBs by 80th day. After 80th day, the young plantlets were shifted back to PPIM towards the development of plantlets with roots and shoots during subsequent cultures. Around 100th day, when tiny roots and shoots started to develop, it was observed that the developmental process in the plantlets emerging from PLBs (Fig. 1j) was slow as compared to those initiating from the protocorms (Fig. 1k). After 100th day, the plantlets were redistributed in many plant tissue culture bottles through continuous subcultures every 20 days interval to ensure further development, providing enough space for the emergence of root and shoot in the plantlet successively. Around five plantlets were shifted to a single plant tissue culture bottle, which provided adequate space for the pseudobulb (thickened stem) of the plantlet to heighten up. Nearly 15–20 plantlets were developed in each culture vessel within a single subculture. Through this platform, from a single capsule, nearly 200–300 young plantlets could be generated within just 80 days of seed culture. The *D. ovatum* plantlets were found to be suitable for transfer to pots for acclimatization after 160 days of seed culture (Fig. 1l). The plantlets of *D. ovatum* exhibited 100% survival during the acclimatization process. After another 20 days, plantlets were finally ready for the ecorestoration experiments (data not shown). The biomass assessments (fresh and dry weights) and their comparisons in proliferated young plantlets/seedlings (100 days old) within a single subculture (within 20 days) indicated that, mostly, there was a significant increase in both fresh and dry weights from 0 to 20th day (Fig. 2).

### Callus induction and biomass comparison

**Fig. 3:**
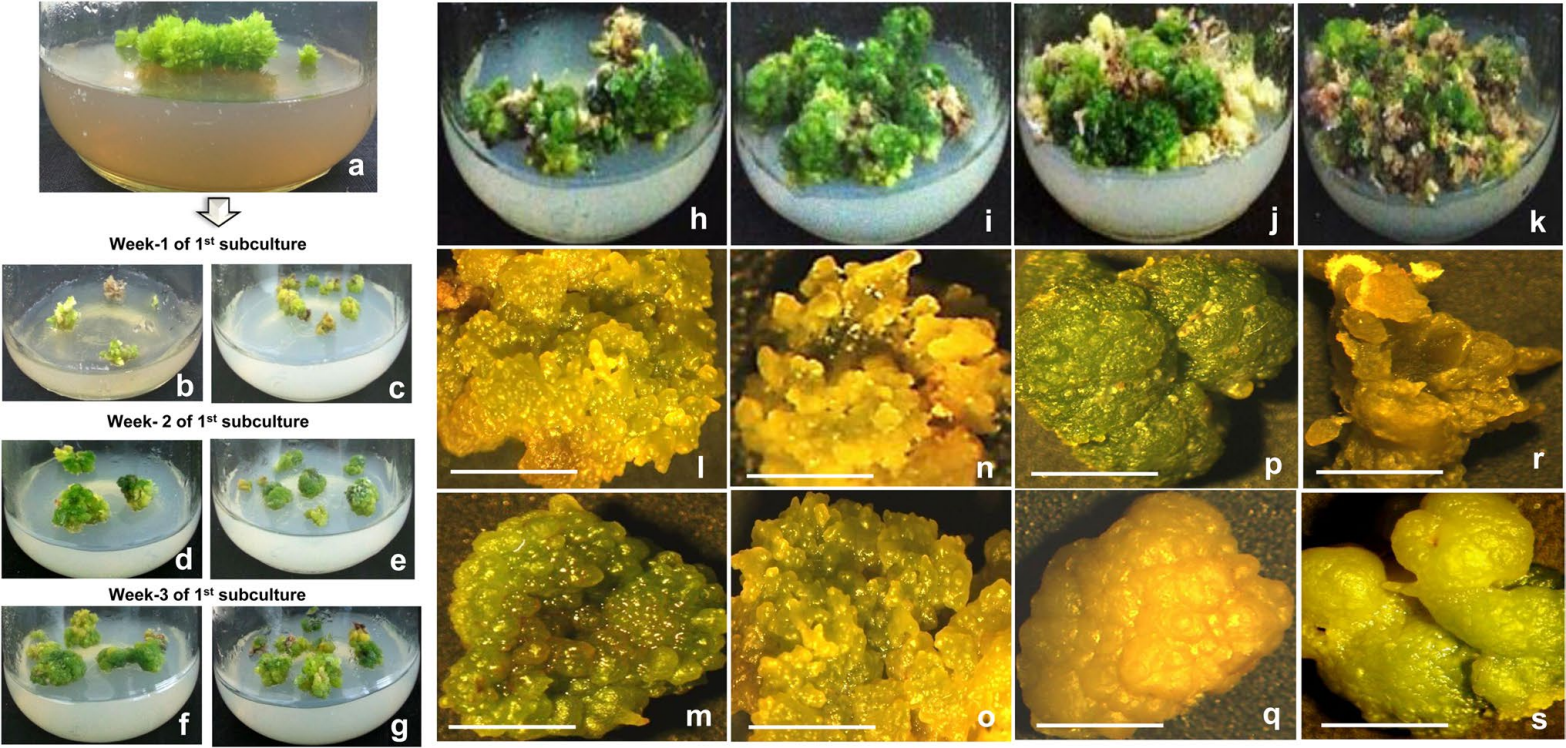
Protocorms and PLBs were used as explants for callogenesis (**a**). Calluses started to develop, when the explants were placed in the half-strength MS medium containing 1 mg /l 2,4-D, 0.5 mg/l 6-BAP in combination with 0.5 mg/l Zeatin and 3% Sucrose, shown in two different culture bottles, during week-1 (**b**, **c**), week-2 (**d**, **e**) and week-3 (**f**, **g**) of 1st subculture. Proliferation of calluses, shown after the third (**h**) fourth (**i**), fifth (**j**) and sixth (**k**) subcultures. Stereomicrographs of embryogenic calli (**l**–**o**), non-embryogenic calli (**p**, **q**), and organogenic calli (**r**, **s**), shown after the fourth subculture (Scale bar = 1 cm)

**Fig. 4:**
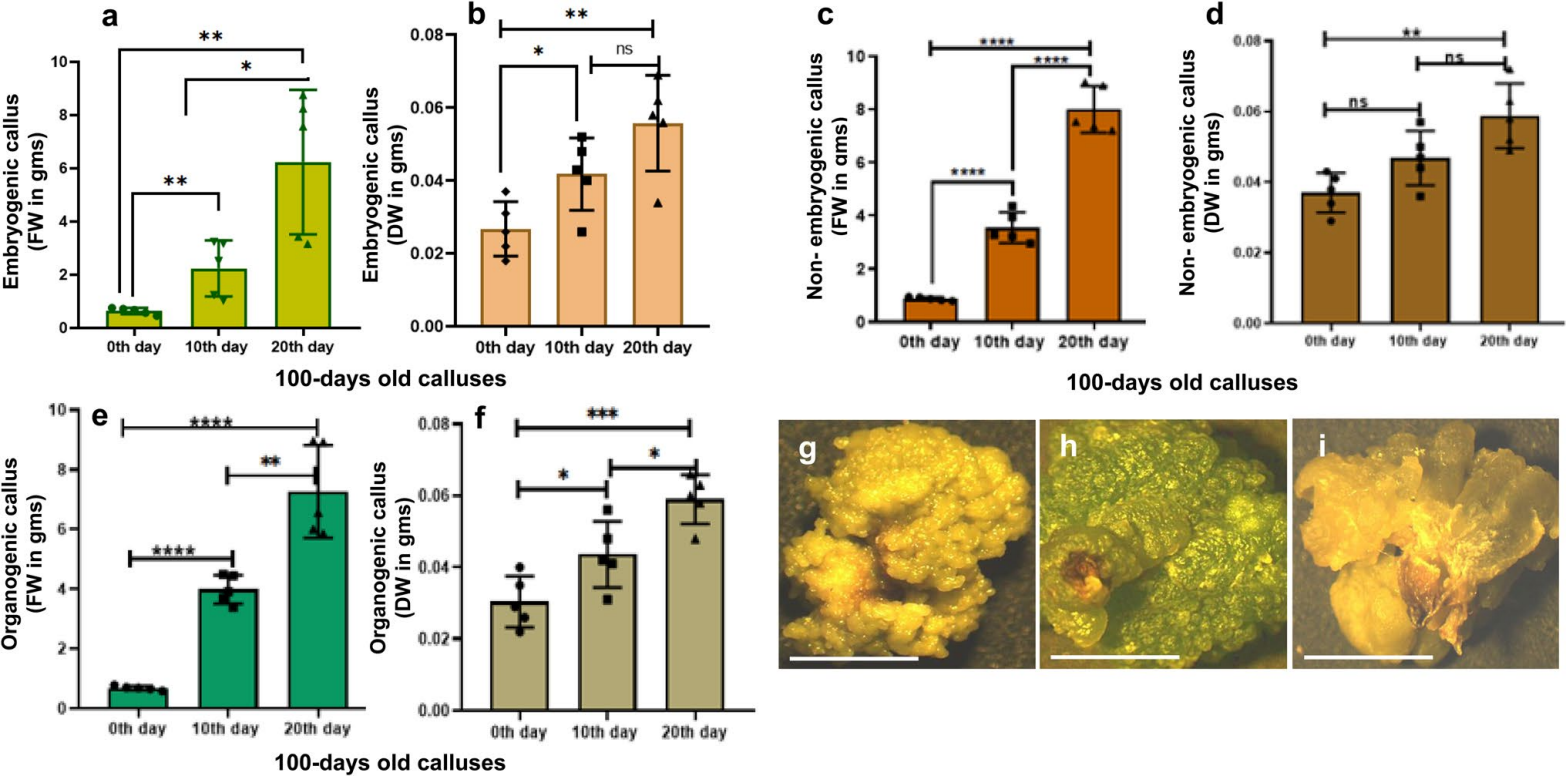
Graphical representations of both Fresh Weight (FW) and Dry Weight (DW) in well-proliferated 100 days old calluses of all three types. FW and DW of embryogenic callus (**a**, **b**), non-embryogenic callus (**c**, **d**) and organogenic callus (**e**, **f**). Each group comprise five replicates. One-way ANOVA with multiple comparisons was performed to obtain the value of significance between the groups. Representative stereomicrographs of embryogenic callus (**g**), non-embryogenic callus (**h**) and organogenic callus (**i**) on the 10th day during a single subculture duration (Scale bar = 1 cm)

Callogenesis was achieved when both protocorms and PLBs (Fig. 3a) were shifted to the half-strength MS medium supplemented with 1 mg/l 2,4-D in conjugation with 0.5 mg/l each of 6-BAP and Zeatin with 3% of Sucrose. At the beginning (during the first subculture), the process of callus growth was slow (Fig. 3b–g). But continuous subculturing resulted in elevated callus biomasses, as they started to proliferate more (Fig. 3h–k). Through this platform, three different types of calluses were obtained viz., embryogenic callus, non-embryogenic callus and organogenic callus. Embryogenic calluses were mostly found to be greenish-yellow, globular and heart-shaped (Fig. 3l–o); whereas non-embryogenic calluses were usually translucent, heart-shaped and they were observed in both green and yellow colors (Fig. 3p, q). Organogenic calluses were found to be yellowish-green in color with the torpedo and cotyledonary shape (Fig. 3r, s). Embryogenic calluses were found emerging faster with more proliferation on the half-strength MS medium in comparison with the other two callus types. All the callus types with enhanced proliferation were achieved around 100 days of callus culture (i.e., after their fourth subculture). Embryogenic calluses were compact primarily, and they formed in globular shapes, but subsequently got enlarged into asymmetric heart-shapes. Oraganogenic calluses primarily developed in an elongated torpedo shape, but gradually got transformed into cotyledonary shape. After the third subculture, the calluses were segregated as embryogenic, non-embryogenic and organogenic. Well-proliferated calluses (100 days old) were used for the biomass estimations and comparisons in all the three callus types within a single subculture duration. In most of the callus types, a significant increase in both fresh and dry weights was observed from 0 to 20th day. The fresh and dry weight of embryogenic callus around the 20th day was found to be lower than the same of non-embryogenic and organogenic callus (Fig. 4). These calluses were maintained further for more proliferation, and an average of 20 gms of each of them was achieved by 200 days of habituation (during ninth subculture) in the medium. Embryogenic and organogenic calluses could form plantlets when they were shifted to the regeneration medium, from which the plantlets with rooted leafy shoots developed gradually.

### Plantlet regeneration and biomass comparison

**Fig. 5:**
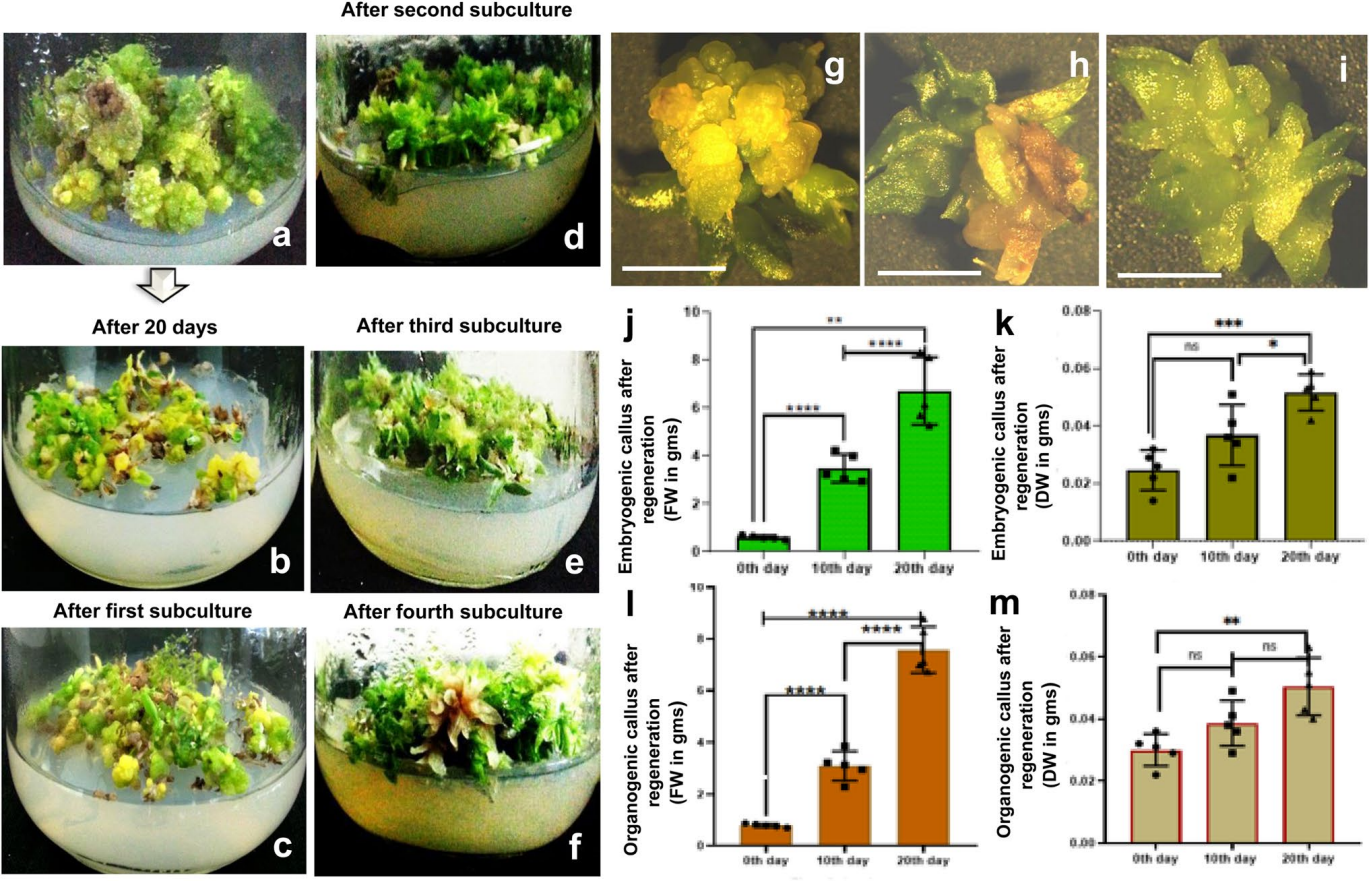
Calluses (embryogenic and organogenic) of *D. ovatum* that were used as explants for plantlet regeneration (**a**). Regeneration from calluses started to develop in 20 days, and when the calluses were shifted to half-strength, MS supplemented with 2 mg/l 6-BAP along with 1 mg/l Zeatin and 5% Sucrose (regeneration medium) (**b**). Growth of regeneration from calluses after the first subculture (**c**), second subculture (**d**), third subculture (**e**), and fourth subculture (**f**), respectively. Stereomicrographs of regenerative plantlets after the second (**g**), third (**h**), and fourth (**i**) subcultures, respectively. (Scale bar = 1 cm). Graphical representations of both Fresh Weight (FW) and Dry Weight (DW) of well-proliferated 100 days old regenerative plantlet developed from the embryogenic callus (**j**, **k**), and organogenic callus (**l**, **m**). Each group comprised five replicates. One-way ANOVA with multiple comparisons was performed to obtain the value of significance between the groups

For the regenerative plantlet formation, well-proliferated 100-day old embryogenic, non-embryogenic and organogenic calluses were taken as explants (Fig. 5a). The regenerative plantlets emerged from both embryogenic and organogenic calluses when they were shifted to a regeneration medium supplemented with 2 mg/l 6-BAP and 1 mg/l Zeatin along with 5% Sucrose. Non-embryogenic calluses were unable to regenerate in this medium. Formation of regenerative plantlets was achieved from both aforementioned callus masses, but the conversion was slow until their first subculture (Fig. 5b, c). By increasing the concentration of Zeatin upto 2 mg/l in the MS basal medium, much proliferation in the masses of callus-derived plantlets was observed during the subsequent subcultures (Fig. 5d–f). The maximum proliferation and growth in the regenerative plantlets were primarily observed after the fourth subculture i.e., after around 100 days. The growth process of regeneration in calluses and their advancement into plantlets was observed as a much slower process in comparison with the growth progression of plantlets derived directly from the seeds. Stereomicrographs of 100 days old callus-derived plantlets that were obtained after the fourth subculture indicated that, they were more sturdy than the seed-derived-seedlings (Fig. 5g–i). After the third subculture, the regenerative plantlets emerging from the embryogenic and organogenic calluses were separated in different tissue culture bottles. Well-proliferated (100 days old) callus-derived plantlets were subjected to biomass estimations and comparisons within a single subculture duration. A significant rise in both fresh and dry weights was visualized from 0 to 20th day (Fig. 5j–m). The fresh and dry weights of these regenerative plantlets were more than the same of plantlets derived directly from the seeds. These callus-derived plantlets were maintained further for extra proliferation, and by 200 days of habituation (during ninth subculture), an average of 20 gms of them was achieved in the medium.

### Moscatilin bioproduction in young plantlets, calluses and regenerative plantlets

**Fig. 6:**
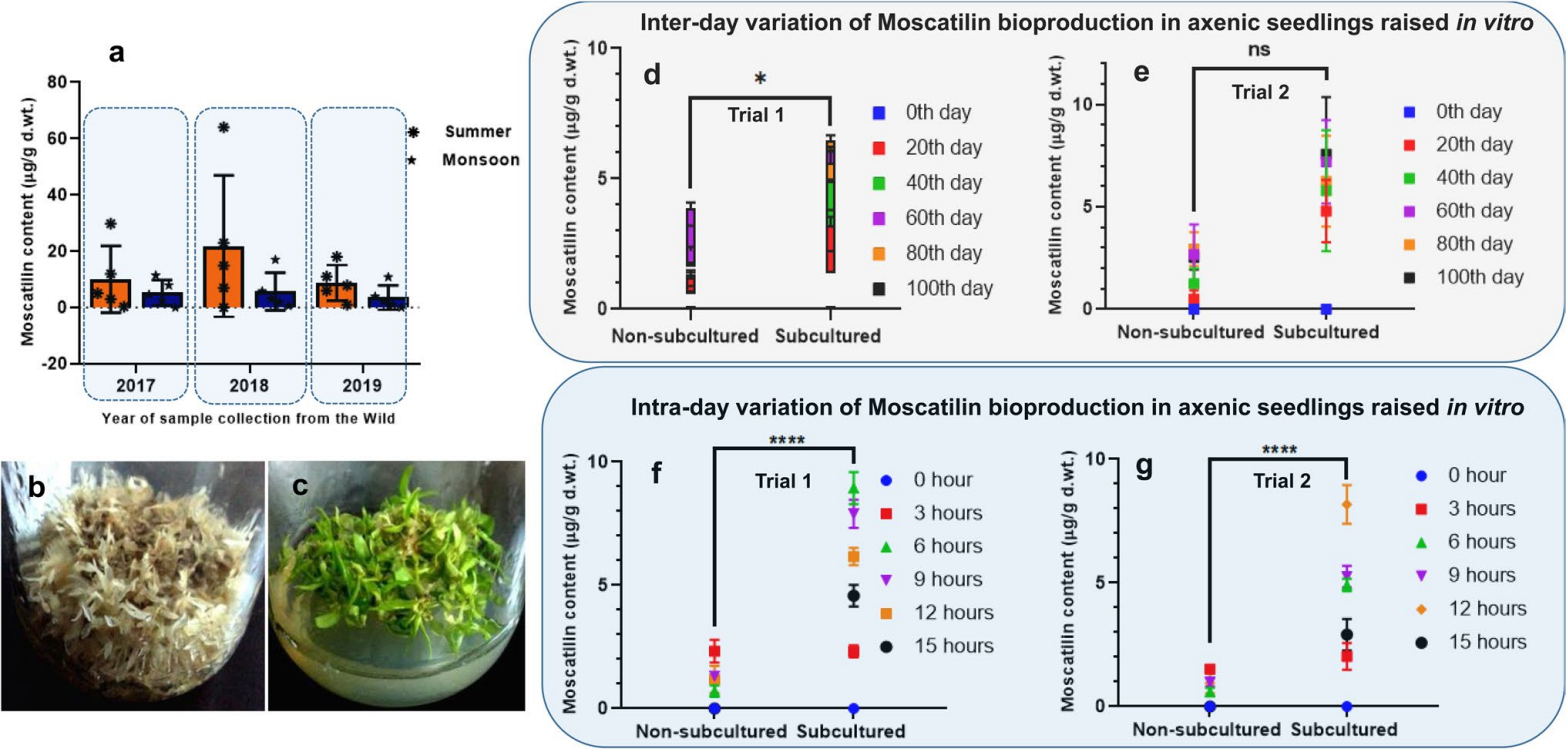
Seasonal influence on the production of Moscatilin in *D. ovatum* whole plants, collected from the wild in the years—2017, 2018 and 2019 during Mid-March [Summer] and Mid-June [Monsoon] (**a**). Each group comprised five replicates. Young plantlets in non-subcultured condition at the 100th day of study (**b**). Regularly subcultured plantlets on the 100th day of study (**c**). Inter-day variations of Moscatilin bioproduction in young seedlings, depicted in the form of two different trials with five replicates in each group (**d**, **e**). Intra-day variations of Moscatilin bioproduction in young seedlings, depicted in the form of two different trials with five replicates in each group (**f**, **g**). One-way ANOVA with multiple comparisons was performed to obtain the value of significance between the groups in both wild and in vitro systems

**Fig. 7:**
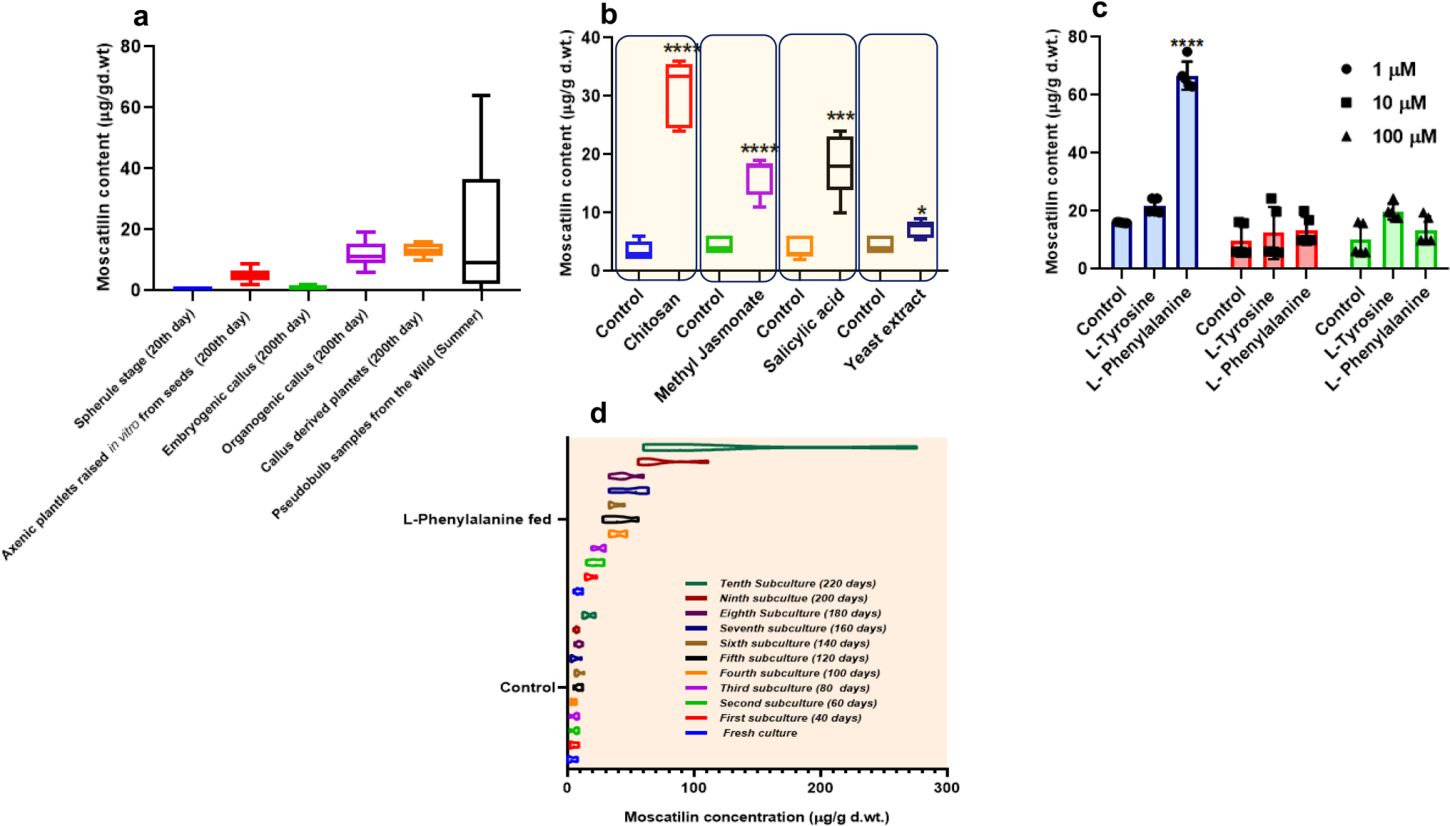
Moscatilin bioproduction comparisons between wild *D. ovatum* plant and all the established in vitro systems (**a**). Box and whisker plots display various elicitors' influence with respect to control on Moscatilin bioproduction from 200 days old regenerative plantlet (**b**). Influence of precursor feeding on Moscatilin bioproduction from 200 days old regenerative plantlet (**c**). Violin plots display the precursor l-phenylalanine's influence (1 µM) on Moscatilin bioproduction from regenerative plantlets, starting from their fresh culture till their tenth subculture (**d**). Each group comprised five replicates. One-way ANOVA with multiple comparisons was performed to obtain the value of significance between the groups

When the plantlets were 100 days old, they were classified as non-subcultured and subcultured, and maintained with or without subculturing for another 100 days. Bioproduction of Moscatilin from these plantlets and the whole *D. ovatum* plant collected from the wild were estimated and compared. All the samples collected from the wild showed inconsistent yield both in Summer [ranging from < limit of detection (LOD) to a maximum of 63 µg/g dry weight (d. wt.) of the extract] and in Monsoon [ranging from < LOD to a maximum of 12 µg/g d. wt. of the extract]. Summer season had comparatively higher Moscatilin content as compared to the same in Monsoon (Fig. 6a). Then the bioproduction of Moscatilin was compared between the young plantlets that were non-subcultured (Fig. 6b) and subcultured (Fig. 6c). Quantification of Moscatilin was done in the form of inter-day (Fig. 6d, e) and intra-day (Fig. 6f, g) variations in non-subcultured and subcultured young seedlings. It was clearly observed that consistent subculturing gave a better yield of Moscatilin as compared to the cultures that did not undergo subculturing. In subcultured samples, the Moscatilin quantity ranged between < LOD to a maximum of 10 µg/g dry weight of the extract, whereas in non-subcultured ones, the lowest was < LOD and their maximum content were below 5 µg/g dry weight of the extract. Moscatilin bioproduction was around twofold higher in subcultured young plantlets in comparison with the non-subcultured plantlets. The observations from inter and intra-day variations in the yields inferred that in vitro young seedlings exhibited stable productivity over wild *D.ovatum* plants. Overall, the yield estimations and comparisons in both wild and in vitro samples indicated that the compound Moscatilin is low-produce by nature. Moscatilin quantity was also estimated during the spherule stage of *D. ovatum,* and it was < LOD (Fig. 7a). This yield was compared with the same getting produced from the young plantlets on 200th day of seed culture, where the maximum was estimated as 8.71 µg/g. The compound yield from calluses till 60th day from day 0 of callus induction was < LOD. For estimation of Moscatilin quantity in callus systems, proliferated calluses on 200th day were considered. The embryogenic callus had Moscatilin productivity ranging from a minimum of 0.34 µg/g to a maximum of 1.98 µg/g dry weight of the extract. In non-embryogenic callus, Moscatilin yield was < LOD. Moscatilin content range was from 6 (minimum) to 19 (maximum) µg/g dry weight of the extract in organogenic callus. The productivity of Moscatilin in the callus-derived plantlets ranged between 10 (minimum) and 16 (maximum) µg/g dry weight of the extract (Fig. 7a). The compound yields in the callus-derived plantlets were relatively consistent as compared to the callus and young plantlet systems. As out of all the three systems, the regenerative plantlet system gave the highest Moscatilin content, and it was chosen to enhance Moscatilin further using precursor feeding and elicitation.

### Impact of elicitors

During the ninth subculture (i.e., 180–200 days old), the callus-derived plantlets were treated with a concentration of 50 mg/l of each elicitor in the liquid half MS medium (PPIM). Chitosan was the most effective elicitor, which gave a Moscatilin yield of 24 (minimum)–6 (maximum) µg/g dry weight of the extract (Fig. 7b). This increase was almost a five-fold rise as compared to their control sets. Salicylic acid treatment gave a yield of 10 (minimum)–24 (maximum) µg/g dry weight, which was a four-fold increment in comparison with their control sets. Methyl jasmonate treatment also exhibited a three-fold increment as compared to the control sets and gave a yield that ranged from 11 (minimum) to 19 (maximum) µg/g dry weight of the extract. The elicitor which had the most negligible impact over the enhancement of Moscatilin was Yeast extract, as it gave a Moscatilin yield between 5.4 (minimum) and 8.9 (maximum) µg/g dry weight of the extract. Also, it did not cause any significant fold increase in Moscatilin content as compared to the control sets. However, long-term treatment with the elicitor Chitosan was toxic to the cells, eventually causing necrosis in plantlets.

### Influence of precursors

**Fig. 8:**
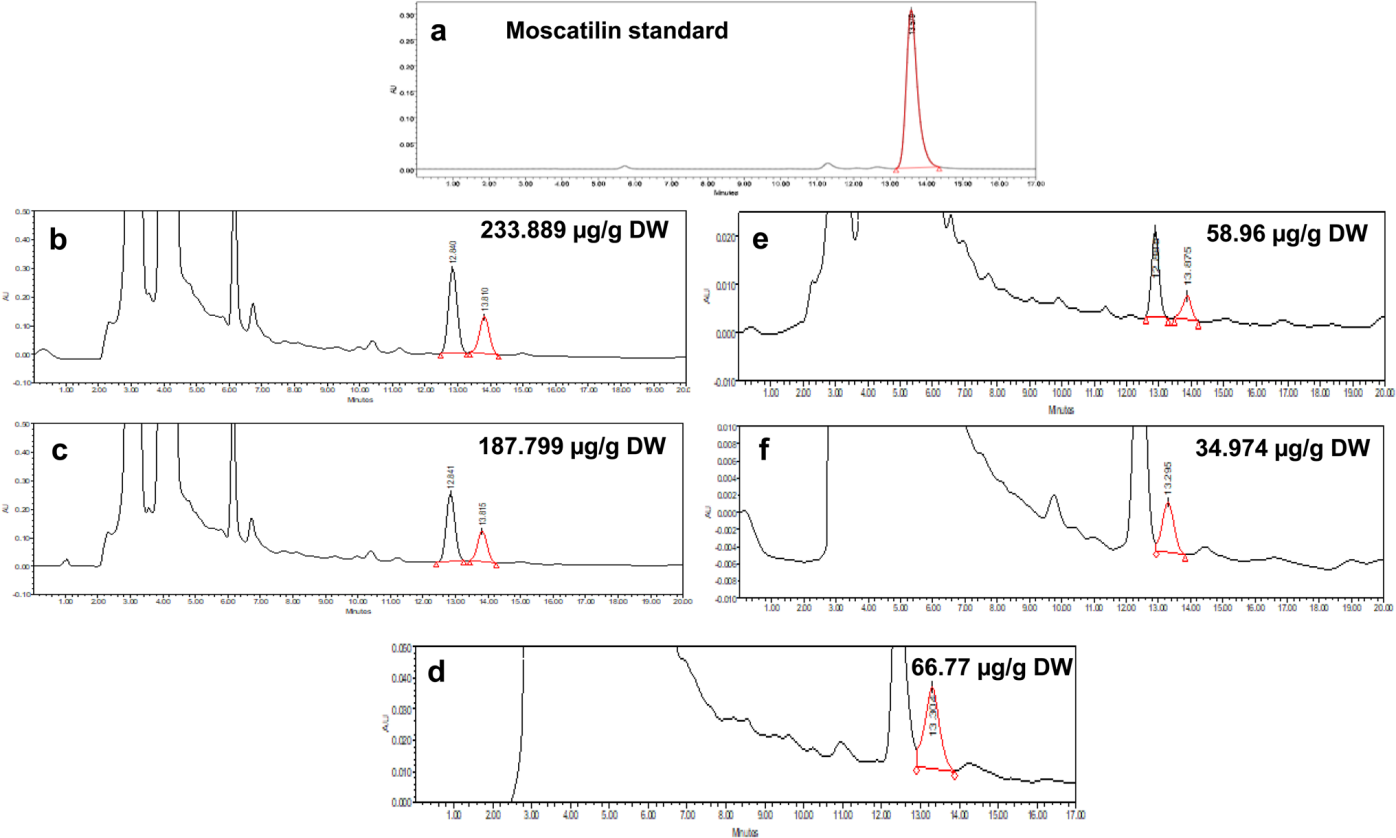
HPLC chromatogram (281 nm wavelength) of Moscatilin as a standard (**a**). Influence of precursor feeding (l-Phenylalanine of 1 µM) on Moscatilin bioproduction from regenerative plantlets on the 200th day (**b**), 150th day (**c**), 100th day (**d**), 60th day (**e**), and 50th day (**f**) of the liquid culture

l-Phenylalanine at 1 µM concentration effectively enhanced the Moscatilin levels in half-strength liquid MS medium (PPIM). The Moscatilin yields from the callus-derived plantlets under the treatment of l-Phenylalanine (1 µM), ranged between 62.98 (minimum) and 74.97 (maximum) µg/g dry weight of the extract. There was approximately a four-fold increment in Moscatilin content after this treatment with the precursor l-Phenylalanine compared to its control sets (Fig. 7c). The Moscatilin yields from the callus-derived plantlets under the treatment of l-Phenylalanine (10 µM), gave a maximum yield of 20.00 µg/g and under the treatment of l-Phenylalanine (100 µM), resulted in a maximum yield of 19.53 µg/g dry weight of the extract. There was just a one-fold increase in Moscatilin content after these two treatments with the precursor l-Phenylalanine compared to the respective control sets. The yield was not appreciable when the concentration of l-Phenylalanine was raised to 10 and 100 µM. l-Tyrosine was relatively an ineffective precursor in all three concentrations. The maximum Moscatilin yields from the callus-derived plantlets under the treatment of l-Tyrosine 1, 10 and 100 µM were 24.399, 19.83 and 24.81 µg/g dry weight of the extract. The treatment under the precursor l-tyrosine could cause a one or a two-fold increase in Moscatilin content compared to the control sets. The feeding of precursors was initially applied to the callus-derived plantlets which were in the ninth subculture (i.e., 180–200 days old). To find the precursor's long-term effect, l-Phenylalanine of 1 µM (that yielded maximum Moscatilin) was fed to the regenerative plantlets in the liquid MS media. Its effect on Moscatilin yield was checked from the fresh liquid culture until the tenth subculture (done every 20 days interval) with the respective precursor treatment. Beyond the fourth subculture, Moscatilin showed a steady rise in the l-Phenylalanine treated regenerative plantlets (Fig. 7d). An occasional spike in Moscatilin content beyond 250 µg/g dry weight of the extract was detected in regenerative plantlets, fed with 1 µM of l-Phenylalanine, during the tenth subculture of precursor testing. A consistent and steady rise of Moscatilin production was witnessed from the third subculture onwards. Few of the Moscatilin yields that were observed on different days of the precursor study were represented in the form of HPLC chromatograms (Fig. 8a–f). Overall, the results from the precursor study indicated that l-phenylalanine is a significant influencer towards Moscatilin bioproduction and this plantlet regeneration system in *D. ovatum* can be efficiently used as a model for bioreactor scale-up of the compound Moscatilin.

During precursor feeding and elicitation studies, the presence of Moscatilin was also analyzed at the extracellular level, but it was primarily found < LOD. Occasionally, 0.5–0.7 µg/g dry weight of the studied compound was detected in the liquid half-strength MS medium (data not shown).

### Identification of Moscatilin in young plantlet, callus and callus-derived plantlet through ESI-QTOF-MS

**Fig. 9:**
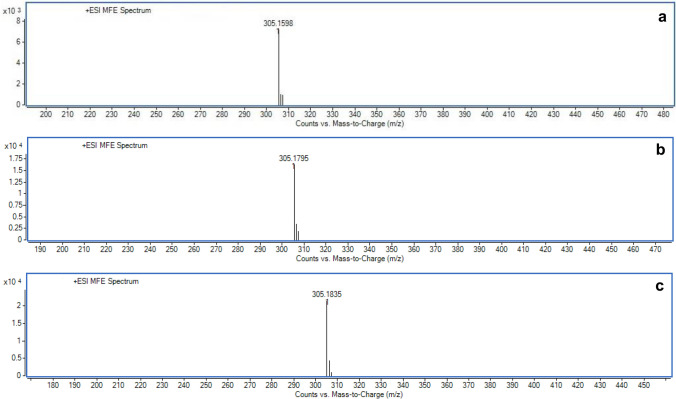
Mass spectra displaying the *m/z* (mass-to-charge ratio) values of Moscatilin as 305.1598 in young plantlet (**a**), 305.1795 in callus (**b**) and 305.1835 in regenerative plantlet (**c**) samples, when run in a positive mode through ESI-QTOF-MS

Moscatilin displayed an *m*/*z* (mass-to-charge ratio) value of 305.1598 in young plantlets through targeted analysis, which was run on a positive mode. A value of 305.1795 and 305.1835 was obtained for callus and callus-derived plantlets, respectively, when they were processed through the mass spectrometer (Fig. 9a–c). The mass values of Moscatilin was compared with the same that exists in the PubChem database, and the compound was confirmed as Moscatilin.

## Discussion

Moscatilin is a bibenzyl derivative (stilbenoid) categorized under the ‘polyphenols’ class of plant secondary metabolites. It has been elucidated in many research studies that polyphenolic compounds such as stilbenoids accumulate more under conditions of biotic (through pathogen attack) and abiotic stress (such as injury, high temperature/radiation, drought, heavy metal, and pesticide), providing greater tolerance to the plants against unfavorable environmental constraints (Sharma et al. 2019). The stilbenoid class of phytochemicals has drawn great attention consistently because it includes a highly studied compound, ‘Resveratrol’ (3,5,4ʹ-trihydroxystilbene). This compound has been identified with many therapeutic properties such as antioxidant, anti-inflammatory, cardioprotective, immunomodulatory, neuroprotective and especially anticancer (Gianchecchi and Fierabracci 2020). Moscatilin is a structural analogue of Resveratrol. The anticancer action of Moscatilin has already been described in the form of cytotoxicity at non-toxic concentrations (Chen et al. 2019), but the final therapeutic dose for this phytochemical is yet to be established. Both Resveratrol and Moscatilin have been reported in a few species of *Dendrobium* (He et al. 2020). Though there are many research investigations on Resveratrol, studies on Moscatilin are minimal. To date, the only source of obtaining Moscatilin is through natural tissues (mostly from *Dendrobium* plants), which are either threatened or endangered. With significant anticancer properties chiefly coming from many threatened *Dendrobium* species, Moscatilin producing tissues requires a biotechnological approach to procure regular supplies in the form of bioproduction and enhancement.

This study showed that the wild plants of *D. ovatum,* which were collected during the summer (recorded temperature: 35–40 °C), had a high content of Moscatilin as compared to the Monsoon seasons (recorded temperature: 25–30 °C). In summer, generally, the orchid plants are not in an active state of growth but are in a reproductive state. During the Monsoon, the plant stays in an active state of growth (vegetative phase). The physical and chemical stresses on the *D. ovatum* plant during summer drought might have prompted the high production of Moscatilin in this case. It has been reported earlier that the biosynthesis of phenolics is positively connected with high light intensity and temperature (Pérez-López et al. 2018). Likewise, it is negatively correlated with rainfall and lower temperature (Tolić et al. 2017). These reasons might have been responsible for causing the high and low yields of Moscatilin during summer and monsoon seasons, respectively. Gross inconsistencies of stilbenoids accumulations in situ are attributed to multiple confounding factors— both biotic and abiotic which work in unison. It is even more challenging to assess their impact on epiphytic species as host-related confounders also come into action (Petter et al. 2021). Ageing, stress and senescence are closely interconnected mechanisms that are again linked to life cycle transitions (Pérez-Llorca and Munné-Bosch 2021; De Souza et al. 2021; Gulzar et al. 2021; Kaminska 2021). Plants in their wild habitat face both ‘progressive’ as well as ‘stress-induced senescence’, notably in summer seasons. Progressive senescence is a slower phenomenon and may reinforce the defence mechanism in plants. Stress-induced senescence is rapid and causes the strengthening of active and rapid defence mechanisms (Pérez-Llorca and Munné-Bosch 2021). These multiple senescence mechanisms could cause remobilization of primary and secondary metabolites—a plausible reason for inconsistent Moscatilin production. Our study showed that Moscatilin production is also linked to the life cycle transitions of *D. ovatum* (vegetative to reproductive states), which could be one of the causes of inconsistencies witnessed in situ.

Tissue culture methods accelerate the mass production of orchids in a short period. Asymbiotic seed culture has been widely applied in *Dendrobiums*, where the plant does not need a fungal partner for seed germination, and this requirement is met by different inorganic and organic nutrients present in the culture medium (Teixeira da Silva et al. 2015a). This asymbiotic method results in a higher percentage of seed germination than the symbiotic mode and is helpful towards germplasm conservation. A single mature capsule of a *Dendrobium* plant holds 2–3 million seeds (Teixeira da Silva et al. 2015a). During seed culture of *D. ovatum* here, first, the spherules (germinated embryo) were formed, followed by the development of protocorms (which gave rise to leaves and pseudobulb) and PLBs that arose from somatic tissues. In these early developmental stages, Moscatilin was mostly found < LOD through RP-HPLC. The seeds from pods are neither wounded nor stressed and presence of very little stress factors at the early stages is the reason behind the inadequate production of Moscatilin. The yields were quantifiable only from the stage of young seedlings/plantlets, which developed during the 80th day of seed culture. The young plantlets that were not subjected to frequent subculture, did not get proper nutrients in the medium and they died sooner. This nutrient deficiency is reflected in the form of non-chlorophyllous nature in plantlets. Moreover, biomass production was insufficient in this system and less quantities of Moscatilin were detected. In young plantlets, that were subjected to frequent subcultures, a gradual ageing was observed. As subculturing resulted in better growth and development of the young plantlets, an ample biomass production was visualized. More Moscatilin content was detected in subcultured young plantlets as compared to the non-subcultured ones. Some secondary plant metabolites demand differentiation of tissues for their secretion and accumulation. The accumulation of Moscatilin in calluses was found lower than the microshoots derived from embryogenic and organogenic calluses. Moscatilin bioproduction was found highest in callus-derived plantlet, followed by young plantlet and then callus. This has indicated that, higher degree cellular differentiation (notably, redifferentiation) is required for the accumulation of higher concentrations Moscatilin. Few studies have already reported that a certain degree of differentiation is significant during the accumulation of a phenolic compound as compared to non-differentiation/dedifferentiation (Palacio et al. 2012). The callus-derived plantlets were more sturdy than the seed-derived plantlets and this could be due to ploidy level changes. And may be these changes resulted in the increased production of Moscatilin in plantlets regenerating from calluses, as ploidy variation has been seen inducing more phenolics in plants (Mei et al. 2020).

Tissue culture platform aids to analyse and help us to arrive at the best influencer of Moscatilin bioproduction. In vitro platforms gave spot-on results highlighting the causes that highly influence Moscatilin accumulation. The present study also indicated that cellular differentiation, polyploidy (through callus regeneration) and biomass increment positively influences Moscatilin accumulation similar to any phenylpropanoid (Mei et al. 2020). Inconsistencies in any one of these factors could be directly correlated with Moscatilin inconsistency. The present study indicated that callus-derived plantlets gave better yield than protocorm derived plantlets. In vitro conditioning is more promising, as it could stabilise Moscatilin production by eliminating the transient confounding factors. Wounding the filamentous seeds is quite impractical, and this resulted in stress-induced senescence in *D. ovatum*. Wounding of seeds was attempted with a few sets of seeds. The wounding caused the necrosis of seeds and embryo destruction (Shakira and Mohd 2021). As conservation of the species is also one of the goals, wounding of seeds was not adopted for the present study as it impaired the study progression.

In this study, seeds were provided with a useful nutrient medium with all the required elements for the growth and development of *D. ovatum *in vitro. This basal medium was half-strength MS, which was very beneficial towards proliferation and regeneration of shoots in cultures along with callus induction. Overall, it was favourable towards tissue differentiation. MS medium is always the most favourable one during *Dendrobium* micropropagation with Agar as a gelling agent (Teixeira da Silva et al. 2015a, b). The use of Zeatin in the MS also has been demonstrated during the cultures of *Dendrobium* (Teixeira da Silva et al. 2015b). Sucrose has always been the best choice as a carbohydrate source, and it is used for acquiring positive carbon balances (Ferreira et al. 2011; Teixeira da Silva et al. 2015a). An optimal percentage of Sucrose is useful towards in vitro shoot growth as it plays a significant role in regulating cell osmolarity. Here, 2% of Sucrose promoted seedling proliferation, 3% Sucrose was useful for callus induction with proliferation, and 5% Sucrose increased plantlet regeneration.

A combination of auxin and cytokinin is always used for the callus induction in *Dendrobiums* (Lee and Chen 2014), and the same has been applied to this study. A study showed that high Sucrose concentration is linked with the phenylpropanoid biosynthesis that promotes intracellular accumulation of stilbene like polyphenols (Ferri et al. 2011). This can be one reason behind the highest Moscatilin yield from regenerative plantlets that utilized 5% of Sucrose. It has also been reported that Sucrose accelerates phenolic synthesis by upregulating many significant genes encoding enzymes, such as chalcone synthase, stilbene synthase and chalcone-flavanone isomerase, that are involved in the associated pathway (Ferri et al. 2011). Enhancement in phenolic content due to Zeatin application was proven in regenerated red cabbage (Ravanfar et al. 2020), and this also forms the basis of present findings related to the Moscatilin yield in regenerated *D. ovatum* plantlets. High Phenolic accumulations mainly demand tissue differentiation, and it has been described earlier that the capacity of an undifferentiated tissue such as callus to produce more phenolic is narrow. But, a rise in phenolic content has been witnessed after the callus has undergone organogenesis, which aligns with the present study (Owis et al. 2016).

As most of the stilbenes are phytoalexins, their synthesis can be well stimulated through biotic and abiotic elicitors. Chitosan and Salicylic acid in the present study functioned as the most effective elicitors towards Moscatilin enhancement in callus-derived plantlets. Chitosan is a natural linear polysaccharide [d-(1,4)-glucosamine polymer], whose role has been vastly reported towards high stilbene accumulation (Xu et al. 2016). It hinders microbial growth, especially fungal infections and activates defence along with lignification processes. Chitosan of concentration 50 mg/l has successfully accumulated high stilbene without any biomass loss (Xu et al. 2016). That is why this concentration was selected for Chitosan and other elicitors in the present study. Studies have precisely specified the upregulation of *STS* (encoding, stilbene synthase), induced after Chitosan treatment (Ferri et al. 2009). High Moscatilin yield through Chitosan in the present study demonstrates that *STS* might have an active role in the Moscatilin biosynthetic pathway. Apart from *STS*, upregulation of *PAL* (encoding, phenylalanine ammonia-lyase) has also been reported upon Chitosan treatment (Singh et al. 2020). Yeast extract, Salicylic acid, and Methyl jasmonate were the other three elicitors of interest towards Moscatilin accumulation. A study has described that Yeast extract causes the upregulation of *PAL* and *CHS* (encoding, chalcone synthase), whereas Methyl jasmonate mostly regulates the transcription of *STS* (Inyai et al. 2020). This again ascertains the stronger role of *STS* over *PAL*, as Methyl jasmonate yielded more Moscatilin than Yeast extract in callus-derived plantlets of *D. ovatum*. The stronger effect of Chitosan over Methyl jasmonate and Salicylic acid has also been proven towards yielding more stilbene (Ferri et al. 2009), which corroborated our findings. According to a study, Methyl jasmonate decreases the biomass by hindering the mitotic cycle in plants through a G1-phase arrest (Mendoza et al. 2018). This may explain lower stilbene yields through Methyl jasmonate treatment as compared to Chitosan in the present study. Towards Moscatilin biosynthesis, a two-stage culture was necessary to raise the callus-derived plantlets of *D. ovatum*, and elicitation treatment was given at the ninth subculture. Long-term treatment with Chitosan was found growth inhibitory, and it turned the regenerative plantlets necrotic. A study has proven the beneficial effects of Chitosan in *Dendrobium* floral production. This study demonstrated that the treatment with Chitosan led to the enlargement of chloroplasts, indicating that chloroplast is one of the target sites of Chitosan (Limpanavech et al. 2008). Incidence of necrosis in the present study showed that chitosan treatment could lead to cell lethality when administered in long-term cultures. Increased chitosan concentration here caused an increase in pigment degradation that was connected with cell death. Chitosan causes an increased concentration of cytosolic Ca^2+^ along with hydrogen peroxide accumulation, eventually leading to cytoplasmic shrinkage and cell death (Hidangmayum et al. 2019). It causes chloroplast swelling and plasma membrane detachment from the plant cell wall (Zuppini et al. 2003). Parsley suspension cultures have been used extensively as models to study the elicitor-based synthesis of phenolics like coumarin derivatives. It is proved that Salicylic acid as an elicitor increased the incorporation of esterified hydroxycinnamic acids into the parsley cell wall. This could have led to enhanced cross-linking polysaccharides for anchoring phenolic polymers that function as phytoalexins (Kauss et al. 1993). Since Salicylic acid enhances Moscatilin content in the cultures (callus-derived plantlets), notably in differentiated cultures, it evidences that Moscatilin possibly plays the role of phytoalexin in *D. ovatum*. It is also shown that chlorinated Salicylic acid functions better than the parent Salicylic acid forms, as it has high permeability and is resistant to cellular enzymes (Tripathi et al. 2019). Methyl jasmonate is a plant growth regulator that plays an essential role in plant development and defence. Since methyl jasmonate is shown to influence the methylerythritol 4-phosphate (MEP) pathway more than the mevalonate (MVA) pathway (Yang et al. 2012). The biosynthesis of Moscatilin in *D. ovatum* could be via MEP. Methyl jasmonate generates reactive oxygen species (ROS), and this oxidative burst regulates the activity of *PAL* and the accumulation of phenolics (Ho et al. 2020). The elicitation experiments of our study inferred that, out of all the elicitors, Yeast extract gave the least yield of Moscatilin, as the compound content was just moderately enhanced in comparison with the control group. Elicitors generally increase the yield of phytochemicals by modulating the gene expressions of a biosynthetic pathway through a unique receptor. Studying the pattern of *PAL* and *STS* expressions mostly might provide substantial clues regarding the molecular action behind the function of elicitors towards Moscatilin biosynthesis in *D. ovatum*. This part of the study will assist in selecting useful elicitors for industrial scale-up in future.

l-Phenylalanine and l-Tyrosine were used for precursor feeding studies, as these two aromatic amino acids are precursors for many phytocompounds, including phenolics, anthocyanins flavonoids, and phenylpropanoids (Yoo et al. 2013). Both these amino acids are final products of the shikimate pathway that serve towards the biosynthesis of phenylpropanoids. Additionally, the role of genes *PAL* and *TAL* (encoding, tyrosine ammonia-lyase) has also been described in phenylpropanoid metabolism (Barros and Dixon 2020). Studies have reported the increased activities of these two genes under various biotic and abiotic stresses, leading to more metabolite accumulation (Lu et al. 2016; Luyckx et al. 2021; Nisar et al. 2021; Sasi et al. 2021). Along with these two genes, the role of *STS* has been specifically related to the accumulation of stilbenes, as a decline in the expression of *STS* decreases its biosynthesis (Jeandet et al. 2012). l-Phenylalanine has been used as a precursor for stilbene biosynthesis, particularly in plant cultures Kiselev et al. (2012), whereas l-Tyrosine has been utilized for the same through metabolic engineering in microbial hosts (Wu et al. 2013). Both Phenylalanine and Tyrosine get converted into p-coumaric acid, where Phenylalanine takes two reaction steps through *C4H* (encoding, Cinnamate 4-hydroxylase) catalysis and Tyrosine just takes one by resulting in this conversion through bifunctional *PTAL* (encoding, Phenylalanine and tyrosine ammonia-lyase) (Feduraev et al. 2020). In the present study, l-Phenylalanine (1 µM) yielded high Moscatilin content, which showed an increasing trend (in comparison with the controls) after consistent subculturing of the callus-derived plantlets. This infers that the exogenous addition of l-Phenylalanine in the liquid MS medium might have served as a substrate for *PAL,* resulting in a higher quantity of Moscatilin. Few reports indicate that *PAL* activity is not always primarily correlated with the accumulation of more phenolic compounds regardless of using l-Phenylalanine as a precursor (Koca and Karaman 2015). Another study indicated that *STS* contributed higher to synthesising phenolics such as stilbenoids over *PAL* (Andi et al. 2019). This implies the combined role of both *PAL* and *STS* in the Moscatilin biosynthetic pathway. To get a clear idea regarding how both l-Phenylalanine and l-Tyrosine regulate the production of Moscatilin-like-stilbene in plants, the gene transcription processes need to be studied. We can assume that a feed-forward mechanism in regulating the biosynthesis in this case. However, this is just a hypothesis, and it awaits to be proven regarding the compound Moscatilin. Our study did not examine the molecular and biochemical mechanisms associated with both precursor feeding and elicitation in the cultures. It is intended towards providing information on Moscatilin bioproduction and enhancement through potential elicitors and precursors by the conservation of threatened *D. ovatum*.

Seeds from a single pod of *D. ovatum,* when cultured, could generate 20 gms of callus-derived plantlet tissues using a three-stage culture shift through PPIM, callogenesis medium and regeneration medium. 20 gms of this plant tissue was generated after 10th subculture, and it gave 12 mg of Moscatilin after flash purification. Currently, the market value of Moscatilin costs $649 per 10 mg. The HPLC purified Moscatilin from the cultures could yield pure 12 mg of the compound, estimated to be $768. This indicates that every pod of *D. ovatum* is worth $768. The input cost (overestimated cost) of the generated *D. ovatum* culture (containing culture medium with its components, PGRs and precursor) with the highest Moscatilin yield is roughly $325. Chemical companies have not undertaken the chemical synthesis of Moscatilin yet due to the lack of stakeholders. Thus, the culture systems reported in the present study can form a baseline for the optimization of bioreactor scale-up, which promises high economic value.

## Conclusion

**Fig. 10:**
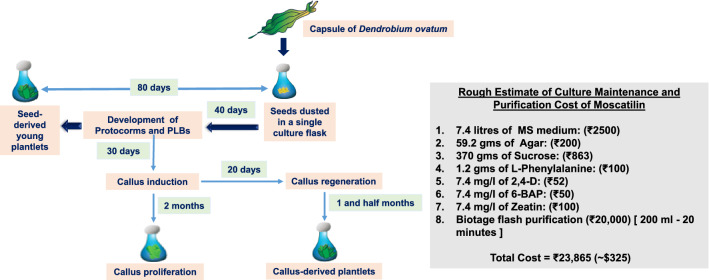
An estimate of culture maintenance of *Dendrobium ovatum* along with the timeline and purification cost of Moscatilin

This research study describes three simple, rapid, competent and economical in vitro tissue culture protocols towards conserving the threatened orchid, *Dendrobium ovatum*. Simultaneously it also demonstrates the bioproduction and enhancement of Moscatilin in vitro from this plant. The seed propagation protocol presents the potential towards the hypergeneration of axenic *D. ovatum* plantlets with high genetic stability, serving as a biomass source. This method can also contribute towards stable Moscatilin yield in vitro, eliminating the influence of environmental stress factors. It can also act as a stage providing the basis for in vitro flowering in plantlets of *D. ovatum*, which can find its purpose in the floriculture trade. Overall, this approach can assist in large-scale propagation, conceiving the opportunities for genetic transformation in *D. ovatum*. The callogenesis approach (with friable calluses) can favor mass cultivation, and it can be rightly utilized for performing cell suspension cultures in moving liquid mediums during bioreactor applications. Out of the three studied tissue culture platforms, the callus-derived plantlet serves as the most commercially advantageous one in terms of the highest bioproduction of therapeutic Moscatilin, employing either l-phenylalanine as a precursor or Chitosan as an elicitor. Therefore, this form can be extended towards the bioproduction and augmentation of other significant bibenzyl derivatives/stilbenoids in *Dendrobium*-like orchids. Altogether, all three in vitro techniques are easily achievable in *D. ovatum* with a shortened growth cycle (only 6 months), in a season-independent mode with reduced cost (Fig. 10).

## Abbreviations

2,4-D: 2,4-Dichlorophenoxyaceticacid
6-BAP: 6-Benzylaminopurine
IUCN: International Union for Conservation of Nature and Natural Resources
LOD: Limit of Detection
MS: Murashige and Skoog
PAL: Phenylalanine Ammonia-lyase
PLBs: Protocorm-like bodies
RP-HPLC: Reversed-Phase High-Performance Liquid Chromatography
STS: Stilbene synthase
